# Supplementary figures and images for: JAK/STAT signaling regulated intestinal regeneration defends insect pests against pore-forming toxins produced by Bacillus thuringiensis
Source: PLoS Pathog. 2024 Jan 18;20(1):e1011823. doi: 10.1371/journal.ppat.1011823 (PMC10796011; doi:10.1371/journal.ppat.1011823)

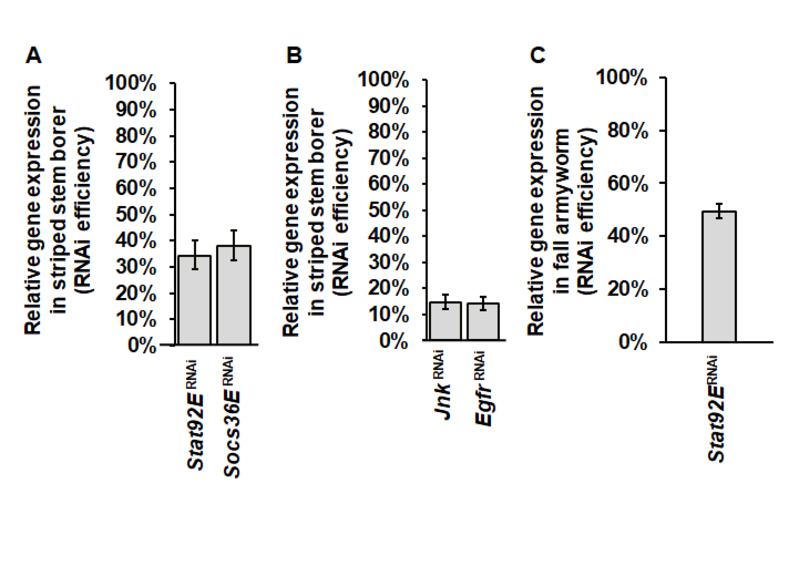

Supplement: S1 Fig — (TIF) [file ppat.1011823.s001.tif]

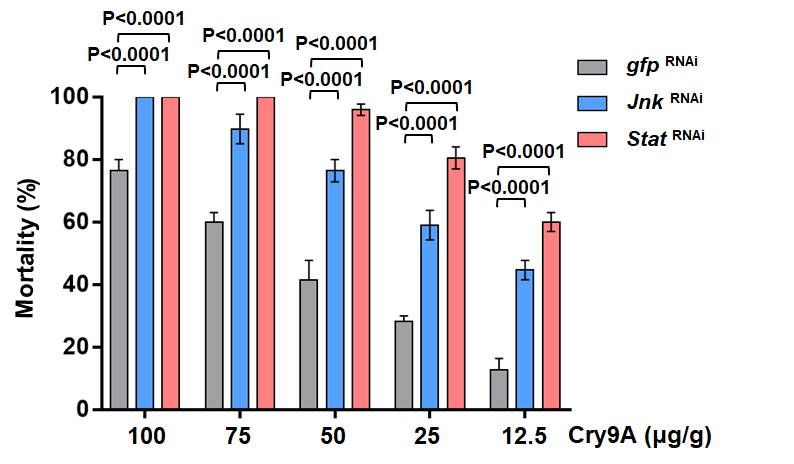

Supplement: S2 Fig — (TIF) [file ppat.1011823.s002.tif]
