# Supplementary material for: JAK/STAT signaling regulated intestinal regeneration defends insect pests against pore-forming toxins produced by Bacillus thuringiensis
Source: PLoS Pathog. 2024 Jan 18;20(1):e1011823. doi: 10.1371/journal.ppat.1011823 (PMC10796011; doi:10.1371/journal.ppat.1011823)
Supplement: Attachment — Submitted filename: Response to Reviewers-submitted.doc [file ppat.1011823.s006.doc]

Dear Editors,

October, 2023

We would like to thank you, and the three expert reviewers for the time spent reviewing our manuscript entitled “JAK/STAT signaling regulated intestinal regeneration defends insect pests against pore-forming toxins produced by *Bacillus thuringiensis*” submitted to *PLOS Pathogens*. We think that the comments of the three reviewers were highly constructive, and helped us to improve the manuscript. Moreover, we have also revised the manuscript according to the *PLOS Pathogens* formatting instructions. We hope you would find the new version of the manuscript suitable for publication. Following are our point-by-point responses to the reviewer’s comments:

**Responses to Reviewers**

**Part I - Summary**

**Reviewer #1:**

*The manuscript proposes the reduction of gut defences to Bt toxins will enhance their effects. The involvement of JNK/JAK/STAT in midgut regeneration is established in Chilo suppressalis and JAK/STAT and JNK silencing is shown to potentiate toxin action through a combination of silencing and inhibition approaches. No effect was seen on Cry9A susceptibility with Egfr silencing. A combined Bt/RNAi XXX was developed, tested and shown to be more effective than Bt treatment alone.*

*This is a very thorough and well carried out study. I have only very minor suggestions for the improvement of the work (see below). There are a few minor errors in English grammar/phrasing but none that obscure meaning.*

We appreciate the reviewer’s positive comments on our work, and we have corrected the errors (see below).

*Reviewer #2:*

*In this manuscript, the authors explore the mechanisms underlying insect resistance to Cry toxins, using striped borers and armyworms. At low toxic doses, they find an increase in intestinal thinning and toxicity when insect JNK or STAT92E is blocked by inhibitors or dsRNA. They treat rice plants with a mix of dsRNA targeting JNK and STAT92E in combination with Bt, and find that the combination has increased efficacy compared to Bt alone. Based on these results, the authors propose a mechanism by which JNK activation in dying epithelial cells triggers Stat activation in stem cells, leading to a regenerative response.*

*The strengths of the manuscript include the importance of Cry toxins, and the need to identify and target survival mechanisms in insects. The authors propose a pathway and show proof of concept in a treatment modality.*

*The weaknesses of the manuscript include the extreme doses used in the treatment, incorrect statistical analysis, missing controls, contradictory LC50 data, and a failure to rigorously test the proposed mechanism by determining if these signaling proteins act in parallel, additively, synergistically, and where they function.*

We appreciate the reviewer’s comments on our work. We have taken in account all your comments to improve our experiments and discussed the questions (see below).

*Reviewer #3:*

*This is an interesting study that investigates the role of healing of the midgut epithelium in pest insect tolerance to Cry toxins from Bacillus thuringiensis. It is heavily motivated by studies in Drosophila and although some conservation in stem cell induction pathways might be conserved across Diptera and Lepidoptera, it is not clear whether there were other target genes from Drosophila that were investigated and not found to have the same effect in the lepidopteran species. Yet the experimental findings from the lepidopteran genes appear to be significant.*

We appreciate the reviewer’s positive comments on our work, and we responded to the reviewer’s questions (see below).

**Part II – Major Issues: Key Experiments Required for Acceptance**

**Reviewer #2: Key experiments needed**

(1) *The authors need to test their proposed model in Fig 6, especially the diverse localization of necessary JNK and STAT92E signaling, and the cell types responsible.*

We agree with the reviewer that localization of JNK and STAT92E signaling and identification of responsible cells is still a missing information. However, these tasks require special experiments that we would like to follow up in the future. We think that our manuscript presents solid data that support our hypothesis shown in figure 6. The importance of our work is that the participation of these pathways leading to midgut cell regeneration after Cry toxin intoxication has not been described before lepidopteran larvae. We improved the discussion and increased clarity in the description of this model. Fortunately these type of studies were published before in another insect, Drosophila, where it was shown that in the ECs, JNK confers stress tolerance and promotes epithelial turnover, and the ligands for the JAK/STAT pathway, such as the cytokines-Unpaired Upd2 and Upd3, are transcriptionally induced in stressed or damaged ECs and EEs, promoting division of ISCs (i.e., Jiang et al., Cell, 2009. DOI: 10.1016/j.cell.2009.05.014; Herrera and Bach, Development, 2019. DOI: 10.1242/dev.167643; Buchon et al., Genes & Development, 2009. DOI:10.1101/gad.1827009; Buchon et al., Cell Host & Microbe, 2009. DOI: 10.1097/01.mcg.0000225688.83206.2c.). We describe these data in the manuscript since they support the model proposed for lepidoptera shown Figure 6. We also edited the discussion saying that more detail information such as the localization of JNK and STAT and identification of cells involved will be relevant to be done in further studies.

(2) *The proposed work does not make it clear if these are additive effects, synergistic effects, or if these are parallel pathways. While Fig 4 suggests JNK may be necessary for Stat signaling, it could be that loss of JNK has indirect effects. For example, if the mode of cell death changes, perhaps key molecules are not secreted to activate Stat/JAK. More rigor in pathway analysis is needed to distinguish between these and other possibilities.*

In our results, we show that the expression of *Jnk* and *Stat* during time and also the effect of silencing *Jnk* that severely affected *Stat* activation. These data suggest that *Jnk* could be involved in the activation of *Stat*. However, we agree with the reviewer comment and we change the manuscript to clearly state that this activation may be direct or indirect and that more studies are required. In *Drosophila* other proteins are involved such as Upd, and homologs of these proteins were not found in the *Chilo suppressalis* genome. Thus, it is possible that other proteins from *Chilo suppressalis* could be playing the function of Upd and this hypothesis requires further research. We improved the discussion of our data and would like to follow up these ideas in future publications.

(3) *It is not clear if the effects observed are due to moving up the lethality curve of Cry9 (i.e. loss of JNK makes insects more sensitive to Cry9), or if these are distinct effects (i.e. JNK is a protective mechanism downstream of Cry9 damage). For example, in Fig 4E, how large are the surviving larvae from a treatment at the LD80 of Cry9A and how much do their intestines thin?*

In this manuscript we were focused in the description of the defense mechanisms against Cry9Aa toxin. For this reason, we selected to work in the analysis of the effect of Cry9A at LD_20_. Figure 4E shows that silencing *Jnk* results in a significant effect on the size of the larvae and thinning of the midgut (Fig 4C). The requested experiment at LD_80_ cannot be performed properly, since this is a high concentration of toxin where the midgut tissue is severely affected, the defense response may not be activated and we could not dissect the midgut tissue from of larvae due to its extreme damage.

(4) *Fig 7 For the insect control assay, the authors sprayed the plants once with a large amount of Bt and dsRNA mix, and then sprayed the insects a second time. This dosing is seems excessive, and it is not clear this would match control in the field. The experiment needs to be repeated with a dose more likely to be used and without spraying the insects themselves. It is concerning that at these doses, only 50-60% mortality was observed in the best case scenario. Also Fig 7E needs the control plants that got no dsRNA with and without insects included and ANOVA for analysis of statistics.*

We have improved this experiment as suggested by the reviewer. We sprayed the plants just once, and reduced the concentration of Bt used to match control in the field. We have changed the third-instar larvae to second-instar larvae. In the new version of the manuscript, we provide a new figure 7, where we added the data about control plants as indicated by the reviewer including the requested statistical analysis.

(5) *Multiple comparisons require ANOVA (with logit or arcsin transformation for percentile data) instead of Student’s t test.*

We have corrected the statistical analysis method: ANOVA analyses were used in comparing the multiple groups.

(6) *In Figure S1, the authors report >50% mortality at both 5 ug/g and 25 ug/g, yet in Fig 1, the LC50 for the 1st instar larvae is ~50 ug/g. This discrepancy needs to be addressed, and full dose-response curves shown.*

In Figure S1, we performed the bioassay with neonates. In contrast with figure 1 where we used 2-day-old larvae to perform the bioassay. It is well known that susceptibility of larvae to Cry toxins is reduced in older instars. This discrepancy has been clarified in the new version of the manuscript.

(7) *Fig 1G, 4D Total Histone H3 is needed as a control for phospho-H3. For Fig 1G, samples should all be re-run on the same gel so they can be compared.*

Total H3 was added in western blot assays in figures 1, 4 and 5 as indicated by the reviewer. The results clearly showed that the concentration of H3 is similar in all conditions.

(8) *Figs 2, 3, 5, a dose-response curve for Cry9 is needed with the RNAi to see how much the kill curve is shifted by JNK or JAK inhibition or by RNAi.*

In this new version of the manuscript we have added new results showing the dose-response curves for Cry9A when silencing *Jnk* and *Stat* in Fig S3. These data showed that silencing *Jnk* and *Stat* increase the mortality of the larvae after Cry9A toxin treatment.

(9) *Fig 5A, a change in mortality from 0.3% to 0.6% is not biologically relevant. Or is this fraction killed, and it is 30% to 60% death?*

The reviewer is right, the change in mortality should be 30% to 60% rather than 0.3% to 0.6%. We have corrected this.

***Reviewer #3****: RNA interference of genes that clearly have other functions other than inducing stem cell proliferation raises the problem of separating a specific effect due to Cry toxin intoxication from other general effects. In Figure 7B, there is a difference in mortality even as early as day 1, before the effects of midgut generation could be seen. How are effects of dsStat93E due to other pathways ruled out?*

We agree with the reviewer, especially in the statement that RNA interference of genes that have other functions may be difficult to interpret. In this new version of the manuscript, we performed negative control where larvae were treated only with Stat-dsRNA without toxin treatment in Fig. 7B. No increasing of mortality was observed after spraying only with Stat-dsRNA, suggesting that RNA interference of this gene did not show general effect on the mortality. However, we still cannot rule out other effects of Stat92E. The defense response may not be induced by single pathway. We may analyze other defense pathways in the future.

**Part III – Minor Issues: Editorial and Data Presentation Modifications**

**Reviewer #1: Minor points:**

(1) *To tie the work firmly to the new pesticidal protein nomenclature, it would be useful if, at least once in the manuscript, the full toxin names for the proteins used were listed. Eg is Cry9A, Cry9Aa1 or another variant. Similarly for Vip3A and Cry1F. This detail may be important in the future to allow comparison with the activities of other Cry9A, Cry1F or Vip3A variants. By specifying the toxin variants used in this work, readers will also be able to understand the D125R mutant more clearly if they wish to find the original, wild-type sequence. If the authors do not currently have official BPPRC names for the proteins used, they can be obtained rapidly by submission of sequences through the BPPRC web pages.*

We added the full name of Bt proteins in the materials and methods: lines 396, 398.

(2)*Line 130 (also elsewhere eg lines 140, 397, 405, 411, 717, 718) -please check throughout: gram should be abbreviated to g not gr*

We have replaced gr with g (gram abbreviation) throughout the revised manuscript.

(3) *Lines 139 (and throughout): the authors use the terms LC20 and LC2. These are somewhat unusual terms (we usually deal with LC50 or LC90) and it would be useful to define them for the reader on first occurrence in the text. The methods section describes the calculation of LC50 but does not mention calculation of LC20 and LC2 values. I assume that these descriptions LC20/LC2 in the text are to be interpreted as concentration required to give lethal effect in 20%/2% of tested insects. It would be useful if their calculation was described. If this is not what is intended by the terms LC20/LC2, please define them and how they were worked out.*

The reviewer is right, LC_20_ and LC_2_ represented protein concentrations that kill insects by 20% and 2%, respectively, we mentioned these in lines 131-134: “When a sublethal dose of Cry9A toxin (200 μg per g of diet) corresponding to a dose that kill 20% of the larvae (lethal concentration value = LC_20_) was used to feed 3rd instar larvae, important changes in midgut tissue morphology were observed.” We have also added the description of calculation method in lines 410-413.

(4)*Lines 154-157: APN has been used previously to measure cell shedding and the method is used here. The description in the result or in the methods does not make clear whether there is a positive or negative correlation of activity with cell shedding. This should be clarified -I assume from the graph that it is a positive correlation (otherwise possible use of APN as a Cry9A receptor and any reduction in activity due to toxin binding may need to be discussed in relation to these results). Fig1F y axis should be labelled as p-nitroanalide released not as cell shedding as p-nitroanalide release is what was actually measured and the units of OD relate to p-nitroanalide absorbance; cell shedding is implied but not measured.*

We have added this sentence into methods: “The higher APN enzymatic activity was the result of an increase in intestinal cell shedding that represented intestinal damage condition. The APN has not been reported as a receptor for Cry9A protein. In these experiments, we used as negative control (Ctrl) a non-toxic Cry9A-D125R mutant protein, which the mutation site is located in domain I helix α3. This mutant lost toxicity but retains the same ability to bind with BBMV as Cry9A.” We have labelled the Fig1F y axis as p-NA release as indicated by the reviewer.

(5)*Fig2B and C: The line for +Cry9A is rather confusing. It appears to be directly beneath bars 1-4 and might be interpreted as referring to those bars. In fact I think it refers to 3-6 and it is approximately under the sloping captions for these bars. Please find a clearer way to annotate*

We have optimized the annotation to make them easier to understand in almost all figures of the manuscript (Fig 1, 2, 3, 4, 5, and 7).

(6)*Line 270: stem striped borer -> striped stem borer*

We have changed stem striped borer with striped stem borer.

(7)*Fig 6 legend, I would suggest changing “Pore-forming toxins from Bacillus thuringiensis …” to “Pore-forming toxins such as Cry9A, Cry1F and Vip3A from Bacillus thuringiensis …”. The involvement of the proposed pathway has not been demonstrated with Bt toxins such as App or Tpp pore-forming toxins, for example, and it would be best not to overgeneralise beyond what has been shown here.*

In Fig 6, the legend has been changed to “Pore-forming toxins such as Cry9A, Cry1F and Vip3A produced by *Bacillus thuringiensis* (Bt) form pores on the apical membrane of larval midgut cells”.

**Reviewer #2:**

(1) *Were dead insects excluded from the analysis of gut thinning? If not, live vs dead insects needs to be separated out.*

The dead insects were indeed excluded from the analysis of gut thinning because it was not possible to dissect intact midguts from dead insects. We have used more accurate expression.

(2) *In the figure legends, it is not clear if 1st or 3rd instar larvae were used for most experiments.*

We have added clear description of the instar larvae used in the different experiments in the legend of almost all figures (Fig 1, 2, 3, 4, 5, and 7).

(3) *The methods state that 500 ug of RNA was used for cDNA synthesis. This seems high. Is this a typo?*

Thanks for pointing out this mistake. We have replaced 500 ug with 500 ng.

(4) *Fig 1A, the full dose-response curves should be shown.*

We have added the full dose-response curves in Fig S3.

(5) *Fig 1C, D, the quantitation does not match the images shown. For example, in 1C the 20 ug dose at 24 h is thinner than the 200 ug dose at 24 h, but 1D shows the 200 ug dose had more thinning.*

In Fig 1D, it can be found that the bars of Cry9A 200 μg and Cry9A 20 μg at 24 h overlap and show no significant differences. Clear differences were only observed at 48 h treatment. We changed the picture in the 20 ug treatment group in the new figure 1C, selecting more representative figures.

(6) *Fig 1H, how were there 3 biological replicates from 15 larvae? Is the RNA from multiple larvae pooled per experiment? If so, how many are pooled?*

Fifteen midguts were dissected from third-instar larvae, these samples were collected together and RNA were extracted. This experiment was repeated 3 times. The sentence now reads: “Thirty 2-day-old larvae or fifteen midguts from third-instar larvae were collected together and mixed in a single sample for analysis of RNAi efficiency and for gene expression determination for each treatment” in Materials and Methods.

(7) *Fig 2, the second labels (Cry toxin or time) do not match the conditions because they are shifted too far to the left.*

We have redrawn this figure.

(8) *Fig 2E, F, the scale should go to 0 not 40.*

We have redrawn Fig 2E, F and Fig 3E, F.

(9) *Quantitation of Fig 4E and 5H are needed, and RNAi larvae without Cry toxin are needed as controls.*

We have redrawn Fig 4E and 5H including the requested control, and we added Fig 4F and 5I with the quantitation of larval length.

(10) *In Fig S2, the Stat92E knockdown is only 50%-70% by RNA. Was there an impact on protein levels? Or could this be an off-target effect?*

In Fig S2, the knockdown efficiency is close to 65% that is relatively high in lepidoptera species by RNAi and was enough to observe a phenotype (i.e., Sun Y et al., Pest Management Science, 2020. DOI:10.1002/ps.5789; Dong X et al, Developmental & Comparative Immunology, 2016. DOI:10.1016/j.dci.2015.09.009; Hui X et al, Insect Molecular Biology, 2011. DOI:10.1111/j.1365-2583.2011.01081.x.).

**Reviewer #3***:*

(1) *Please include the Lepidopteran species names in the keywords*

We have added *Chilo suppressalis*, *Spodoptera frugiperda* in the keywords.

(2) *line 73, induces, not induce*

We have replaced “induce” with “induces”.

(3) *twice the following phrase is used, " increased the larvae susceptibility to the treatment with Cry9A ". This is misleading because the susceptibility to Cry9A, an intrinsic property of the species and strain, is not changed. Mortality may have increased or growth may have decreased, but the susceptibility to Cry9A has not changed. "susceptibility to the TREATMENT with Cry9A" adds to the ambiguity. Please choose use a more objective phrase.*

We have rewritten this sentence in all the places. The sentence now reads: ... increased the larvae mortality to the treatment with Cry9Aa”.
